# The Reinforced Inner Tube

**Published:** 1909-08-28

**Authors:** 


### Motoring Notes.

### THE REINFORCED INNER TUBE.

This invention, which has recently attracted considerable notice on account of its many advantages, is well worth the attention of the medical motorist, and, indeed, of all who wish to obtain the maximum of wear out of their covers. The tube, which is an American invention, differs from the ordinary form in the fact that it is reinforced with an inner lining of high grade Sea Island cotton fabric (two-ply in small sizes, three-ply in the larger sizes) covering the portion of the tube that usually blows out, pinches, and punctures. The tube thus does not rely for its support upon the outer cover, but has an auxiliary one of its own. At the rim side or base of the tube there is a narrow strip of strong pure rubber to allow the necessary expansion and contraction, making it possible for the tube to adjust itself readily to the cover. The tube is claimed to be reinforced to such an extent as to add 40 per cent. to the strength of the tyre without extra weight or lessening the resiliency, and to prevent blow-outs, rim cutting, pinching, and many other of the troubles experienced with ordinary tubes, whilst the makers state that the reinforced tube is sufficiently strong to sustain the weight of the car while running on a damaged cover. In order to demonstrate the advantages of the new tube, some comparative tests were made some time ago in the presence of a large number of motorists and other interested spectators. An ordinary inner tube was first inserted in an outer cover with a large hole in it, and inflated to a pressure of about 40 lbs. The car to which the tyre was applied was driven a short distance, when the inner tube burst with a loud report. One of the reinforced tubes was then inserted in the same damaged cover, and the car was taken for a short run in the town, the tube still remaining intact when the vehicle returned, although it had been blown up to a pressure of 62 lbs. before starting. The advantages of this inner tube over the ordinary variety are apparent to all, the most important being the fact that old covers which are impossible of repair for use with the ordinary tube and which are usually deposited on the scrap-heap may, by use with the reinforced tube, be repaired and give a very considerable mileage. The Royal Automobile tests have proved that there is no loss of resilience, and that it possesses this quality in precisely the same degree as the best-known standard tubes. The club tests have also shown that covers can be run over 1,000 miles with large bursts, so it is obvious that with such bursts repaired a very considerable mileage can be obtained from an old cover by means of this invention. It is put on the market by the Reinforced Inner Tube Company, of 218 Shaftesbury Avenue, London, W.C., who will be pleased to forward full particulars to applicants.

